# Real-Life Outcome of Lupus Nephritis with Current Therapies: Study Protocol of a Multicentre Observational Study

**DOI:** 10.31138/mjr.33.2.263

**Published:** 2022-06-30

**Authors:** Maria Pappa, Maria Kosmetatou, Antonia Elezoglou, Kyriaki Boki, Pinelopi Konstantopoulou, Charalampos Papagoras, Alexandros Garyfallos, Dimitrios Vassilopoulos, Prodromos Sidiropoulos, Petros Sfikakis, Dimitrios Boumpas, George Bertsias, Maria Tektonidou, Antonis Fanouriakis

**Affiliations:** 1 First Department of Propaedeutic Internal Medicine, National and Kapodistrian University of Athens, “Laiko” General Hospital, Athens,; 2 Rheumatology Unit, Fourth Department of Propaedeutic Internal Medicine, National and Kapodistrian University of Athens, “Attikon” University Hospital, Athens,; 3 Department of Rheumatology, “Asklepieion” General Hospital, Athens, Greece,; 4 Rheumatology Unit, Sismanogleio General Hospital, Athens, Greece,; 5 Department of Rheumatology, “G. Gennimatas” General Hospital, Athens, Greece,; 6 First Department of Internal Medicine, University Hospital of Alexandroupolis, Democritus University of Thrace, Alexandroupolis, Greece,; 7 Fourth Department of Internal Medicine, Aristotle University of Thessaloniki, Thessaloniki, Greece,; 8 Clinical Immunology-Rheumatology Unit, 2^nd^ Department of Medicine and Laboratory, National and Kapodistrian University of Athens, Athens, Greece,; 9 Rheumatology and Clinical Immunology, University of Crete Medical School and University Hospital of Iraklio, Iraklio, Greece

**Keywords:** lupus nephritis, novel therapies, belimumab, voclosporin, systemic lupus erythematosus

## Abstract

Lupus nephritis (LN) affects a significant proportion of patients with systemic lupus erythematosus (SLE) and is characterised by increased morbidity and mortality. The updated joint EULAR/European Renal Association-European Dialysis and Transplant Association (ERA-EDTA) recommendations for the management of LN have set as target of therapy the optimisation (preservation or improvement) of kidney function, accompanied by a reduction in proteinuria of at least 25% by 3 months, 50% by 6 months, and below 500–700 mg/g by 12 months (complete clinical response). It is currently unknown what proportion of Greek patients with LN reach these proposed targets with the current available treatments. At the same time, recent successful phase 3 trials have led to the approval of both belimumab and voclosporin for the treatment of patients with LN and have steered discussions as to whether the “induction-maintenance” paradigm should be substituted by an early combination treatment for all patients. To inform future therapeutic decisions and facilitate the positioning of these new drugs in the therapeutic algorithm of LN, the current study protocol aims to map the unmet needs in the treatment of LN in Greece, by quantifying the proportion of patients who attain the recommended treatment targets in everyday clinical practice.

## Background and AIMS OF THE study

Lupus nephritis (LN) affects a significant proportion (20–60%) of patients with systemic lupus erythematosus (SLE) and is accompanied by significant morbidity.^1^ To facilitate physician decisions and homogenise patient care, the joint EULAR/European Renal Association-European Dialysis and Transplant Association (ERA-EDTA) recommendations for the management of LN were recently updated.^2^ According to these, the aim of LN management should be the “optimisation (preservation or improvement) of kidney function, accompanied by a reduction in proteinuria of i) at least 25% by 3 months, ii) 50% by 6 months, and iii) a UPCR target below 500–700 mg/g by 12 months (complete clinical response). These targets were set based on post-hoc analyses of randomized controlled trials and observational studies, which have shown that reductions in proteinuria within the first 3, 6 or 12 months are associated with favourable long-term outcomes in LN. In particular, post-hoc data from the MAINTAIN and Euro Lupus Nephritis trials showed that proteinuria values 0.7 and 0.8 g/day, respectively, had the best predictive value for a serum creatinine < 1.0 mg/dl at 7 years.^3,4^

Following a series of unsuccessful trials, the field of LN management has witnessed major advances over the past year, which have led to the approval of two new drugs, belimumab and voclosporin. In the BLISS-LN trial, belimumab was compared to standard-of-care in patients with new-onset or relapsing proliferative or membranous LN.^5^ At 2 years, significantly more patients in the belimumab group met the primary endpoint of renal response (43% vs. 32%; odds ratio 1.6), and a lower risk of renal-related events or death (hazard ratio 0.51). Shortly thereafter, voclosporin, a newer, more potent calcineurin inhibitor, was tested on top of mycophenolate mofetil (MMF) in patients with LN, versus MMF alone.^6^ Patients in the VCS group had significantly higher and earlier response rates compared to the placebo group (complete renal response rate at 52 weeks 41% vs. 23%; OR 2.65). These studies, along with the also successful, yet smaller study of obinutuzumab,^7^ have steered vivid discussion regarding the place of new agents in the therapeutic armamentarium and whether the long-standing paradigm of “intense induction – milder maintenance” treatment of LN will eventually be replaced by combination treatments from the beginning.^8–10^ This novel approach is supported by the fact that LN is by definition a severe SLE manifestation and carries considerable disease- and treatment-related complications; moreover, the percentage of complete response in patients with LN who receive standard-of-care treatment in LN clinical trials is consistently below 40% at 6–12 months, meaning that the majority of patients have incomplete response. Nevertheless, it is not clear whether this suboptimal response rate in clinical trials mirrors real-world evidence, wherein changes in the treatment or medication dosing are more flexible compared to the stringent environment of a clinical trial. In this regard, a recent real-life study in 104 patients from two European centres, with frequent use of cyclophosphamide, reported a more than 90% rate of complete response at the end of the ∼3-year follow-up (almost 70% at 12 months), a finding clearly different than clinical trial results, although the Caucasian predominance in the cohort should also be considered.^11^ To better understand and conceptualise the position of these new drugs in the therapeutic algorithm of LN, it is imperative to know the magnitude of the “unmet need” in LN treatment, i.e., how many patients fail to reach the aforementioned targets with standard-of-care therapy in real-life settings. In this regard, it is currently unknown what proportion of patients with LN in Greece attain complete or partial response at 3, 6, or 12 months following treatment initiation, as well as at long-term follow-up. To fill this gap, the current study aims to map the current unmet needs in the treatment of LN in Greece, in order to inform future therapeutic decisions in the face of upcoming treatments.

## METHODS

### Study design and inclusion criteria

The study will consist of two parts: i) a retrospective cohort, and ii) a prospective cohort part, and will be conducted in various hospital in Greece (Laikon General Hospital, ‘Attikon’ University Hospital, “Asklepieion” General Hospital, General Hospital “Sismanoglio:, “G. Gennimatas” General Hospital, all in Athens, Greece, as well as University Hospital of Iraklio, Crete, Hippokration University Hospital of Thessaloniki, University Hospital of Alexandroupolis). Ethics Committee approval will be obtained by all participating centres.

The following patient inclusion criteria will be applied:

1. histologically confirmed LN (histologic class IIIA/IIIA+C, IVA/IVA+C, V or mixed III–IV + V [ISN/RPS 2003]), within the previous 5 years (retrospective part), as well as all new LN cases following the launching of the study (prospective part).
2. Regular follow-up, ie, at least every 3 months for the first year following diagnosis and every 3–6 months thereafter, until the last visit (retrospective cohort) or completion of 3 years follow-up (prospective cohort).

Demographic, clinical and laboratory data will be collected at baseline and every 3 months for the first year, and every 3–6 months thereafter until completion of the study, as outlined below.

## REGISTRY VARIABLES

For each patient, the following variables will be collected at inclusion visit (initiation of treatment):

- Demographics (sex, nationality, date of birth)
- Date of SLE diagnosis and date of LN diagnosis
- SLE classification criteria (ACR 1997,^12^ SLICC 2012,^13^ EULAR/ACR 2019^14^)
- Global activity indices (SLEDAI-2K,^15^ Physician Global Assessment [PhGA]^16^)
- Irreversible organ damage (SLICC/ACR Damage Index)^17^
- Comorbidities (Rheumatic Disease Comorbidity Index [RDCI])^18^
- Histologic type of LN and other histologic characteristics (eg, activity and chronicity index)
- Immunologic variables (ANA, anti-ds DNA, anti-Ro, anti-La, anti-Sm, anti-RNP, C3, C4, direct Coombs, anti- β2GPI IgG/IgM, anti-aCL IgG/IgM, lupus anticoagulant)
- Treatment initiated for LN (previous, ongoing): detailed record of medications (main treatment, concomitant disease treatments) and administered forms/dosage
- ○ For patients included retrospectively detailed information on the induction therapy and changes in treatment at 3- or 6-month intervals, as described below
- Previous treatments received for SLE (ie, prior to LN diagnosis)

Following treatment initiation, at time points 3,6,9, 12, 15,18, 24, 30, 36 months, the following variables will be documented:

- eGFR (MDRD calculated)
- Level of proteinuria (24h collection or UPr/Cr in urine spot)
- Haematuria (if urinalysis available)
- SLEDAI-2K disease activity index
- SLICC Damage Index (SDI), PhGA
- Drug changes (as well as reason for change), including:
  ○ Any change in immunosuppressive medications (including antimalarials)
  ○ Change in glucocorticoid dosages o Other drugs (eg, antihypertensives)

## OUTCOMES UNDER STUDY

After documentation of the above variables, in patients with adequate data, the following outcomes will be assessed:

1. Percentage of attainment of the therapeutic targets set by EULAR/ERA-EDTA, especially the percentage of complete renal response at 1 and 2 years, in a real-life setting
2. Relating to the above, efficacy and safety of different therapeutic regimens for initial and sequential treatment of LN
3. Incidence of renal flares over time (nephritic and proteinuric)
4. Incidence of chronic kidney disease (CKD) and end-stage kidney disease (ESKD) at the end of follow-up
5. Prognostic factors of complete response and, contrary, of no response
6. Degree of control of extrarenal disease activity over time
7. Irreversible damage accrual over time (SDI)

### Data entry and collection

Each participating centre will be granted access to the electronic platform (registry) with unique credentials, in order to enter their own patient data, as previously performed in similar multicentre real-life studies endorsed by the Hellenic Rheumatology Society and Professionals Union of Rheumatologists of Greece.^19,20^

### Statistical analysis

An export file of all patient data from participating centres will be obtained for statistical analysis (months 31–36). Descriptive results on demographics will be reported and the proportion of patients with partial or complete clinical response at the various timepoints will be calculated. Attainment of complete response will be associated with baseline clinical and demographic characteristics, as well as immunosuppressive regimens used and dosing of glucocorticoids. Rates of renal flares and respective predictive factors will also be evaluated.

### Timeline

February 2022: Anticipated study approval from the Ethics Committees of all participating centres

March 2022–March 2023: Retrospective part of study: Patient chart review for patients with incident LN within the previous 3 years who have adequate data, as outlined above

March 2022–March 2025: Prospective part of study: Patient inclusion, monitoring, and data documentation

December 2022 and December 2024: Presentation of preliminary study results in the National Congress of the Hellenic Society of Rheumatology and Professionals Union of Rheumatologists of Greece

**Figure 1.**
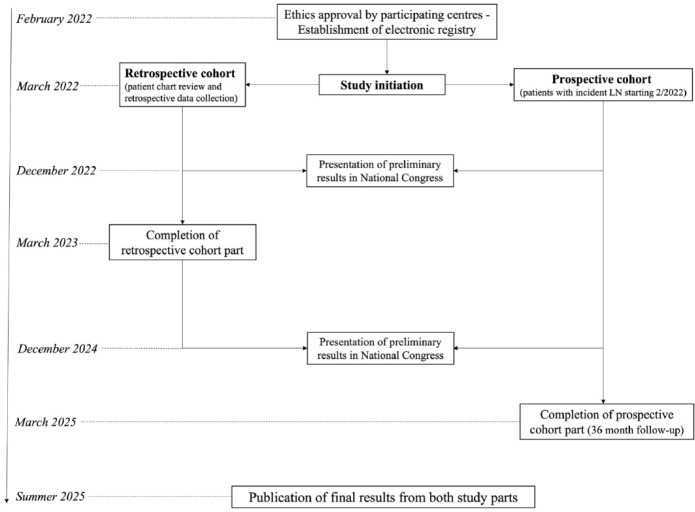
Flow diagram and major milestones of the study protocol.

Total study duration: 36 months (3 years) (**Figure 1**)

## ANTICIPATED RESULTS AND PROJECT SIGNIFICANCE

With the recent advent of new therapies for LN, it is important to identify the proportion of patients who do not respond optimally to current state-of-the-art therapy in real-life clinical settings, and thus would potentially benefit from new treatments. Identification of LN phenotypes that may benefit more and the efficacy of new agents in patients with poor prognostic factors is important.^21^ While an early-on combinatorial therapeutic approach sounds appealing in a disease with significant morbidity and considerable impact on patients’ quality of life and survival, there remains a possibility that some patients may be overtreated by combination therapies. Regarding belimumab, the cost of treatment is also an issue that must be taken into account.

Based on the above, this multicentre observational real-life study will attempt i) to quantify the magnitude of the current unmet need in LN therapy in Greece, and ii) to identify factors associated with poor response or, on the other hand, good response to specific immunosuppressive agents, in order to guide future decisions and help in the positioning of novel treatments in the therapeutic armamentarium.
